# How Folded Brains Evolved in Mammals

**DOI:** 10.1371/journal.pbio.1002001

**Published:** 2014-11-18

**Authors:** Janelle Weaver

**Affiliations:** Freelance Science Writer, Carbondale, Colorado, United States of America

One hallmark of human evolution is the expansion of a part of the brain called the neocortex, which is involved in high-level functions such as sensory perception, language, and conscious thought. Despite considerable progress in the study of brain size evolution, the adaptive mechanism that evolved along certain mammalian lineages to produce a large and folded neocortex has not been clear.

**Figure 1:**
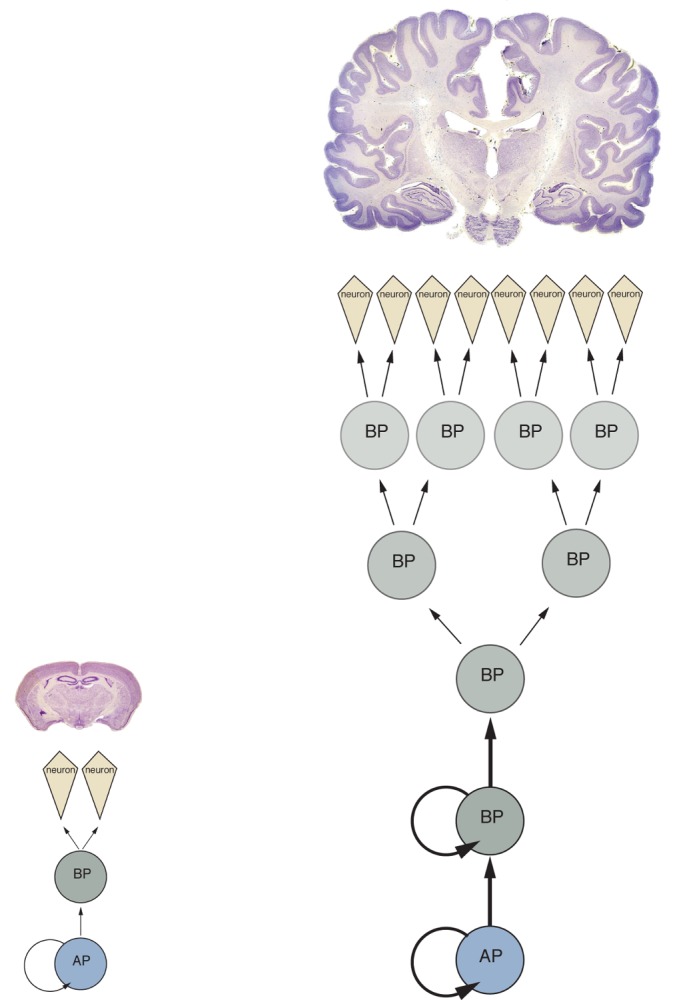
Two distinct neurogenic programs, differing by (i) the absence (left, mouse) or presence (right, human) of basal progenitors capable of symmetric proliferative divisions and (ii) the duration of neurogenesis (vertical dimension), segregate mammalian species into two principal neuroanatomical groups. AP, apical progenitor; BP, basal progenitor. (Images of mouse and human brains from brainmuseum.org. Images not to scale.)

In a study published this week in *PLOS Biology*, Eric Lewitus and Wieland Huttner of the Max Planck Institute of Molecular Cell Biology and Genetics and their collaborators addressed this question by analyzing 37 physiological and life-history traits for 102 mammalian species using phylogenetic tools and mathematical techniques. They found that mammalian species form two distinct groups that are separated by a threshold value of cortical folding, and neocortical expansion beyond that threshold requires symmetric proliferative divisions of basal progenitor cells during development (Figure 1). According to the authors, the study reveals that not all mammalian brains are built the same and that evolutionary biology can be useful for elucidating principles of developmental biology.

In the new study, the researchers first measured cortical folding of species sampled from every mammalian order by analyzing brain tissue sections and collecting values from the scientific literature. Using phylogenetic tools to capture the evolutionary history of cortical folding, they found that the Jurassic-Period mammalian ancestor may have had a relatively large brain with a folded neocortex, but cortical folding has since been highly variable across species. Notably, mammals constitute two main groups with cortical folding values above and below a specific threshold corresponding to 1,000,000,000 cortical neurons. For example, dolphins and foxes exceed this threshold, while mice and rabbits do not. Besides having a greater number of neurons, species with a high degree of cortical folding accumulate on average 14 times as much brain weight per gestation day compared with species with low cortical folding values. Importantly, differences in cortical neuron number between species within each group can be explained by differences in the length of the gestation period rather than by different developmental processes.

To examine the developmental mechanisms accounting for differences in cortical folding across species, the researchers used a mathematical model of the birth of cortical neurons. They found that evolutionary gain or loss of proliferative potential in a brain structure called the subventricular zone is an essential mechanistic determinant of neocortical expansion. In particular, an increase in the proliferative potential of basal progenitor cells is an adaptive requirement for traversing the evolutionary cortical folding threshold. But it is still not clear whether basal progenitors capable of undergoing these symmetric proliferative divisions are an ancestral mammalian trait that was subsequently lost in certain species over evolutionary time, or whether, instead, this trait evolved independently in different lineages.

When the researchers analyzed life-history traits, they found that the two groups of species on either side of the threshold have adapted to their environments differently. While species with low values of cortical folding are associated with narrow habitats and small social group sizes, species with a high degree of cortical folding tend to spread across wide habitats and form large social groups. The findings suggest that life-history traits and ecological factors may exert selection pressure on neocortical evolution.

The study paves the way for future research on the adaptive influence of ecological and life-history traits on species above and below the threshold. Moreover, future research on the conservation of genomic regions regulating the capacity of basal progenitors to undergo symmetric proliferative divisions in different mammalian species may reveal whether this mechanism for neocortical expansion has evolved independently in distantly related species or is the product of a deep homology in mammalian cortical development.

**Lewitus E, Kelava I, Kalinka AT, Tomancak P, Huttner WB (2014) An Adaptive Threshold in Mammalian Neocortical Evolution.** doi:10.1371/journal.pbio.1002000

